# Novel resistance mechanisms of a wild tomato against the glasshouse whitefly

**DOI:** 10.1007/s13593-016-0351-4

**Published:** 2016-02-22

**Authors:** Thomas McDaniel, Colin R. Tosh, Angharad M. R. Gatehouse, David George, Michelle Robson, Barry Brogan

**Affiliations:** 1 School of Biology, Newcastle University, Devonshire Building, Newcastle-upon-Tyne, NE1 7RU UK; 2 Stockbridge Technology Centre, Cawood, Selby, North Yorkshire, YO8 3TZ UK

**Keywords:** *Trialeurodes vaporariorum*, *Lycopersicon pimpinellifolium*, EPG, Plant-pest interactions, Breeding

## Abstract

The glasshouse whitefly, *Trialeurodes vaporariorum*, is an important pest of many crop plants including tomato, *Solanum lycopersicum*. Many wild tomato species exhibit a higher resistance to whiteflies. Therefore, locating the source of this enhanced resistance and breeding it into commercial tomato species is an important strategy to reduce the impact of pests on crops. Here, we assessed the pest resistance of *Lycopersicon pimpinellifolium* by comparing oviposition and feeding data from *T. vaporariorum* on this wild tomato species with data collected from a susceptible commercial tomato, *S. lycopersicum* var. ‘Elegance’. The location of resistance factors was examined by use of electrical penetration graph (EPG) studies on these tomato species. Results show that whiteflies preferentially settled on the commercial tomato more often in 80 % of the replicates when given free choice between the two tomato species and laid significantly fewer eggs on *L. pimpinellifolium*. Whiteflies exhibited a shorter duration of the second feeding bout, reduced pathway phase probing, longer salivation in the phloem and more non-probing activities in the early stages of the EPG on the wild tomato species compared to the commercial tomato. These findings evidence that a dual mode of resistance is present in this wild tomato against *T. vaporariorum*: a post-penetration, pre-phloem resistance mechanism and a phloem-located factor, which to the best of our knowledge is the first time that evidence for this has been presented. These findings can be used to inform future breeding strategies to increase the resistance of commercial tomato varieties against this important pest.

## Introduction

One of the foremost arthropod pests of glasshouse crops, and in particular, tomatoes, is the glasshouse whitefly, *Trialeurodes vaporariorum* Westwood. This phloem-feeding, cosmopolitan, homopteran pest damages plants in three ways. Whiteflies extract sap from phloem during feeding and reduce the nutrients available to the plant for growth and reproduction (Byrne 1991). They also produce a sticky excreta called honeydew which supports sooty mould growth on the plant, limiting its photosynthetic potential and causing aesthetic damage to fruits, reducing their commercial value (Inbar and Gerling 2008). Finally, whiteflies transmit damaging viruses via their saliva, such as the *Tomato chlorosis* and *Tomato infectious chlorosis viruses* (Jones 2003).

Under glasshouse production, the foremost whitefly control method is the use of biocontrol agents, including *Encarsia formosa* Gahan. This parasitoid wasp oviposits into the immobile nymph stages of the whitefly, with the subsequent emerging larvae using the nymphs as a food source (Gorman et al. 2007). Whilst these agents are a moderately effective control measure, the method has several limitations. For one, multiple releases, often on a weekly basis, are typically required to manage whitefly numbers. Deployment of biocontrol is thus labour-intensive, also requiring that wasps are dispensed rapidly after arrival for maximum efficacy. Secondly, and perhaps more importantly, biocontrol agents alone are not always sufficient to reduce whitefly numbers below acceptable thresholds, with biocontrol often breaking down under extreme pest pressure, or in the face of natural movement of hyperparasitoids into the system. In these instances, it is necessary to deploy chemical pesticides as a ‘second line of defence’ to redress balances between pest and parasitoid or to replace biocontrol where this has failed to function due to the appearance of a fourth trophic level. In this respect, pesticides remain a key component of current glasshouse crop production (George et al. 2015).

Several different synthetic pesticide classes are used to control whitefly, including neonicotinoids, pyrethroids, pyrethrins and spirocyclic phenyl-substituted tetronic acids (Fera 2015). However, effective use of conventional chemical pesticides is becoming increasingly difficult due to whiteflies evolving resistance to active ingredients, tightening legislation restricting availability of approved products, and consumer concerns regarding chemical persistence in the environment, which may impact upon non-target and beneficial species (Karatolos et al. 2010). Owing to these shortcomings of current control methods, alternative methods of reducing whitefly impact on crop plants are currently being sought, with significant recent effort being directed to investigating the potential of biopesticidal and biorational products against this pest (George et al. 2015). Whilst such work has merit in potentially expanding pesticide availability, such ‘reduced risk’ products are not free from limitations, which, depending on product types, can include reduced residual activity, environmental sensitivity and variable efficacy (George et al. 2015). Free from such limitations, an alternative, complementary and at least equally promising approach is to increase crop resistance to whitefly pests through incorporation of genes from more resistant wild tomato species into commercial varieties.

Due to its status as a crop plant of global importance, the cultivated tomato, *Solanum lycopersicum* Linnaeus, has undergone extensive selection to enhance its desirable traits (Bas et al. 1992). This selection process has potentially left the cultivated tomato bereft of the genetic variation required to allow it to cope with a range of environmental and biological stresses, including attack by *T. vaporariorum* (Sim et al. 2011). Therefore, attempts have been made to increase the innate genetic resistance of the cultivated tomato. Crossbreeding methods for breeding genes into commercial plants from wild relatives are an important means of increasing plant resistance to various pests, diseases and stresses: they are under no regulatory scrutiny and are generally well accepted by consumers, unlike genetic modification methods. Wild relatives are often much more resistant to pest attack and have consequently been used in these interbreeding programmes as well as in genetic engineering as gene sources. Several studies have demonstrated the success of this approach to increase plant resistance to the sweet potato whitefly, *Bemisia tabaci* Gennadius (e.g. Morales (2001); Carabali et al. (2013) amongst others), though similar work on *T. vaporariorum* is less prevalent.

Relatively little is known about the molecular responses of plants to phloem-feeding insects, such as whitefly, compared to chewing insects. Whilst most work on whitefly resistance mechanisms has been conducted on *B. tabaci* and not *T. vaporariorum*, the mechanisms are thought to be similar between the two species (Toscano et al. 2002). Due to the highly specialised feeding method employed by this guild of insects, whereby the insect’s stylet negotiates the intercellular space to penetrate the phloem, the defensive response of plants is more akin to that observed in response to pathogenic infection (Zarate et al. 2007). The signalling pathways involved in the plant’s response to whitefly have been studied but evidence for the prevalent pathway is mixed, with jasmonate- and ethylene-responsive signalling pathways proposed to be more important in some studies (e.g. Puthoff et al. (2010)) and salicylic acid being suggested in others (e.g. Zarate et al. (2007)). The only R (resistance) gene in tomato which has been shown to interact with any whitefly species to date is the *Mi-1.2* gene (which encodes a protein with putative coiled-coil nucleotide-binding site and leucine-rich repeat motifs; Bhattarai et al. (2007)) which has been shown to confer resistance to *B. tabaci* in tomato, as well as the root-knot nematode and potato aphid (Nombela et al. 2003). RNA transcripts of jasmonate and ethylene-responsive pathogenesis-related (*PR)* proteins, such as the glucanase *GluB*, the chitinase *Chi9* and *Pathogenesis-related protein-1*, have been shown to accumulate in infested tomato leaves in response to feeding by *B. tabaci* and *T. vaporariorum* nymphs, indicating a role for these proteins in tomato whitefly resistance (Puthoff et al. 2010). Volatile organic compounds may reduce whitefly impact on tomato (Guo et al. 2013), mainly via repellence but also via potential toxic effects (Bleeker et al. 2011). Glandular trichomes on leaf surfaces, particularly type IV and type VI (Firdaus et al. 2013), have been shown to physically ensnare whitefly (Toscano et al. 2002) and exude deterrent or toxic chemicals such as acyl sugars (de Resende et al. 2009). Other physical methods are also important in determining host susceptibility, such as cuticle and epidermis thickness (Toscano et al. 2002). Proteins present in the phloem sap, of which a large proportion of those that have been characterised are predicted to be stress- or defence-related, may also affect whitefly feeding behaviour (Kehr 2006).

*Lycopersicon pimpinellifolium* (L.) Miller is the most closely related wild species of tomato to the commercial tomato *S. lycopersicum*. It has been used previously as a source of genes for hybridising with *S. lycopersicum*, this being facilitated by *L. pimpinellifolium* producing red fruit and the relative ease with which the two species hybridise (Kazmi et al. 2012). *L. pimpinellifolium* has been used to improve several traits in the commercial tomato, including improved seed abiotic stress resistance (Kazmi et al. 2012) and increased vegetative tissue salt tolerance (Foolad and Chen (1999); Foolad (2007)). Many studies have investigated the ability of wild tomato species to resist *B. tabaci*, e.g. Firdaus et al. (2012), Rodríguez-López et al. (2011) (which both included accessions of the wild tomato species considered here), Firdaus et al. (2013) and de Resende et al. (2009). Work to elucidate the source of whitefly resistance in wild tomato species has also been attempted. Studies include comparing the strength of resistance to *T. vaporariorum* of wild accessions and commercial tomato species (Bas et al. (1992); Lei et al. (1999)) and comparing wild accessions with inter-specific hybrids of commercial and wild species (Rodríguez-López et al. 2011). Other studies have looked to compare resistance in commercial tomato varieties to other *T. vaporariorum* host species (Lei et al. 2001). However, to the best of the authors’ knowledge, this work presents the only comparative data on *T. vaporariorum* resistance in *L. pimpinellifolium* compared to a commercial tomato variety, *S. lycopersicum* var. ‘Elegance’.

With the above in mind, the aim of the current study was to assess *L. pimpinellifolium* for resistance to *T. vaporariorum* and to investigate the specific resistance mechanisms present in this wild tomato species. These data will add to the body of knowledge on wild tomato varieties that potentially possess enhanced resistance to *T. vaporariorum* and provide some of the only evidence comparing the commercially used ‘Elegance’ line of tomato to the wild tomato, *L. pimpinellifolium*. In expanding current knowledge of whitefly behaviour on *L. pimpinellifolium*, this work further aims to provide insight into the mechanism of resistance in this wild species.

## Materials and methods

### Insects

**Fig. 1:**
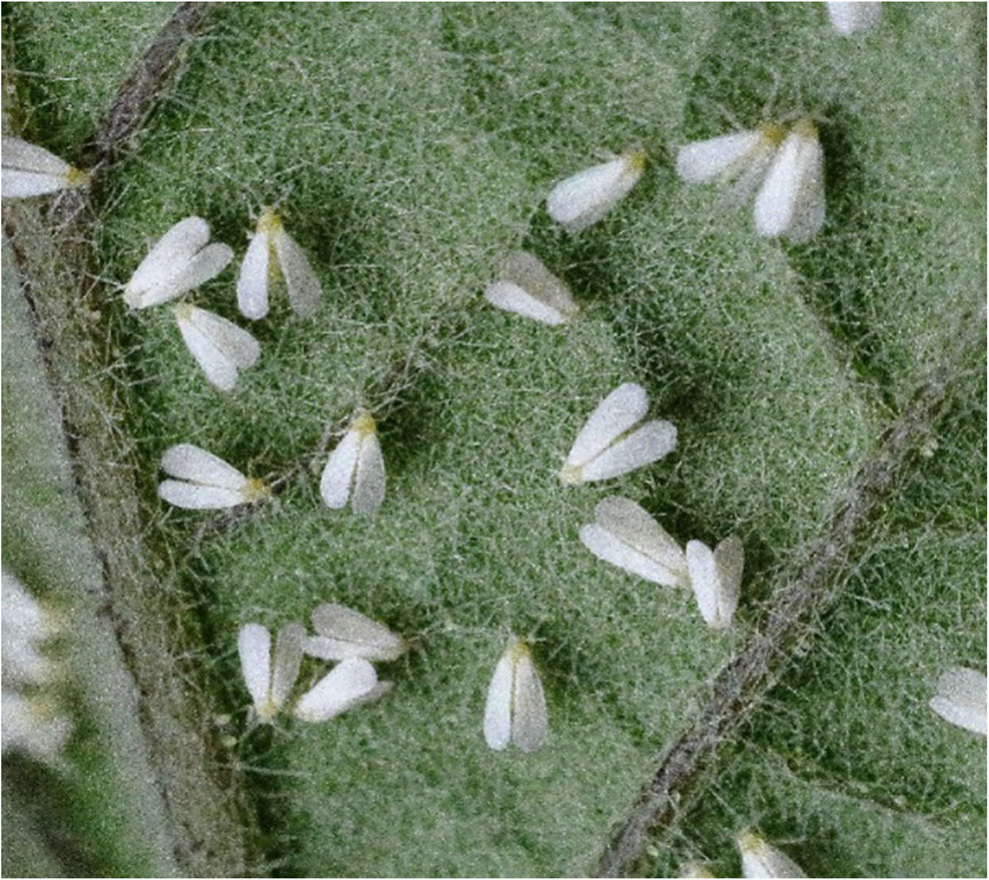
*Trialeurodes vaporariorum* feeding on aubergine

*T. vaporariorum* whiteflies were taken from a mixed-age colony maintained in a laboratory at Newcastle University (UK) on aubergine (*Solanum melongena* ‘Moneymaker’) under 16 h light/8 h dark cycle and constant 20 °C temperature conditions (Fig. 1). This colony was originally obtained from a laboratory culture at Rothamsted Research, first collected in 1960 in Kent originating on French bean plants.

### Plants

Commercial tomato seeds (*S. lycopersicum* Mill., ‘Elegance’ Cat. E/12/11, Batch 0113479253) were obtained from Monsanto, and *Lycopersicon pimpinellifolium* seeds were obtained from Magic Garden Seeds Ltd. (product code LYC09). All plants were grown from seed in Clover Multipurpose Compost (http://www.cloverpeat.co.uk/CLOVER-RETAIL-COMPOST-1.html) in 9-cm-diameter and 8.7-cm-deep pots, at a density of one plant per pot. All plants were grown at a distance of approximately 60 cm from a 400-W Son-T bulb housed in a Harrier HR400SH 400-W lamp under a 16 h light/8 h dark cycle and a temperature regime of 25 °C during the light period and 20 °C during the dark period, synchronised with the light regime that all other experiments were conducted under. Plants were liberally watered before and during the experimental period and used for all assays at the 3–5 true leaf stage.

### Free choice assays

The settling preference of *T. vaporariorum* for the two tomato species was quantified, with whiteflies having free choice between the commercial ‘Elegance’ cultivar and *L. pimpinellifolium*. For each repeat of the experiment, six plants (3 Elegance, 3 *L. pimpinellifolium*) were placed into a 20-L transparent Perspex tank with an open mesh top and were spaced 3 cm apart. Whiteflies were caught using a mouth pipette (a length of rubber tubing with a pipette tip on the end) then placed in a small petri dish and anaesthetised with CO_2_ for 90 s before the petri dish was placed in the cage. In this way, simultaneous release of whiteflies was achieved. Whiteflies of equal gender mix were placed into the cage and allowed free settling choice over the course of 24 h; the number of whiteflies used was 15 males and 15 females for four runs and 40 males/40 females for the fifth run. Numbers were increased for the fifth replicate as high mortality had been observed in earlier runs. After 24 h, the number of settled whiteflies on each plant was recorded. The experiment was conducted under a 16 h light/8 h dark cycle and a constant temperature regime of 20 °C. The differences in settling behaviour were analysed using Pearson’s chi-squared test, with expected values representing an even distribution between plant species after the number of dead whiteflies were removed.

### No-choice assays

**Fig. 2:**
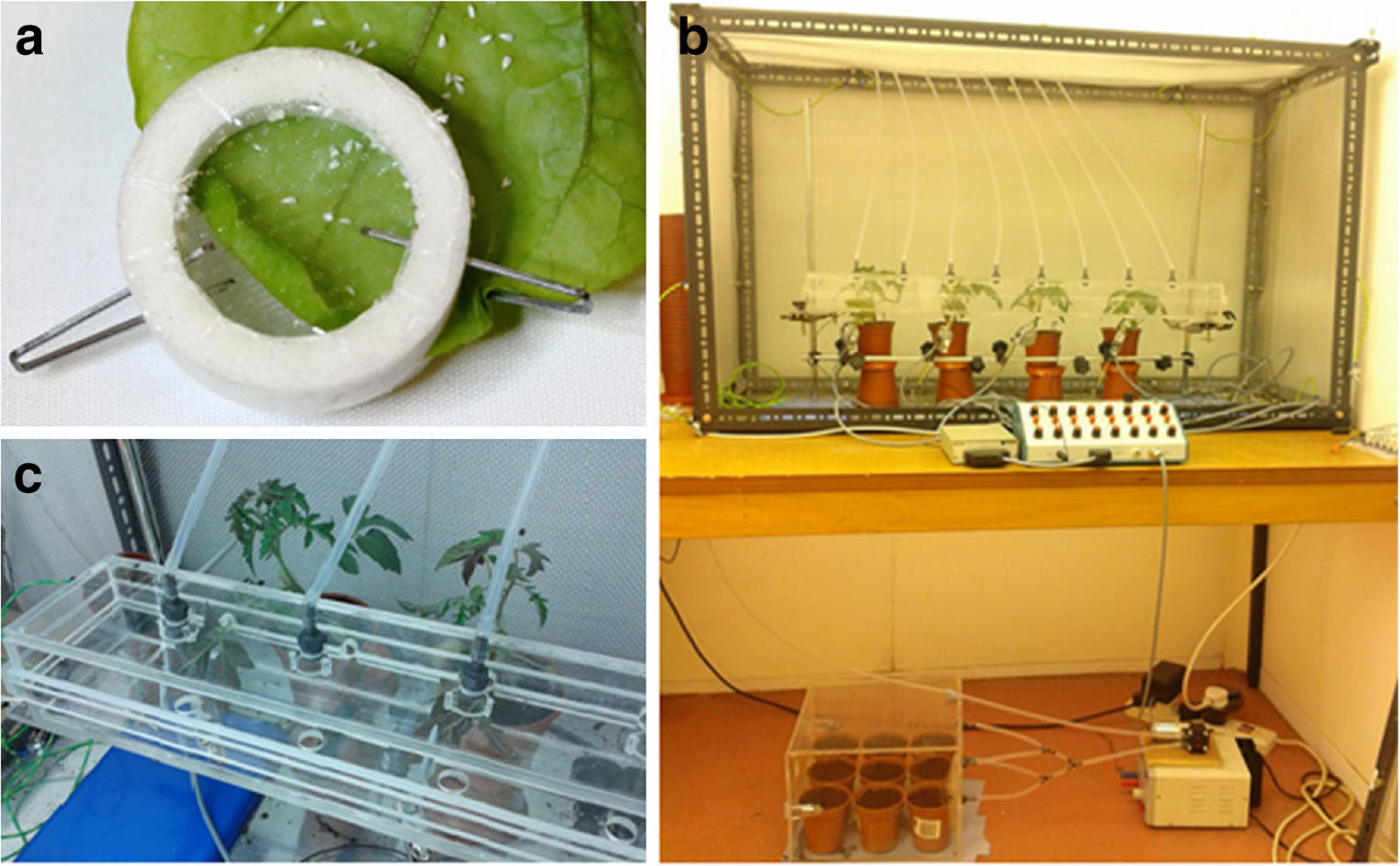
**a** Clip cage used in no-choice and video trials. **b** Electrical pentration graph (EPG) equipment used for EPG studies. **c** Detail of volatile delivery box and positioning of plants for EPG studies

The second behavioural measure taken was the rate of oviposition of whiteflies in a no-choice situation. A single female whitefly was placed on the second apical leaflet of a tomato plant in a small clip cage and left for 72 h, with the plant placed inside a cage (as previously used). The clip cage consisted of two foam rings, with clear acetate over opposing sides of the rings, which could be closed over a leaf and secured using two staples. This allowed the whitefly in the experiment to move on and off the plant as well as between the abaxial and adaxial surfaces of the leaf (Fig. 2a). After 72 h, the clip cage was removed and the leaf was analysed at low magnification (×3) to count the number of eggs laid by the whitefly on both sides of the leaf. Whitefly survival was also recorded, with 17 replicates completed for ‘Elegance’ and 19 for *L. pimpinellifolium*. The data were analysed using the Mann-Whitney *U* test due to deviations from the normal distribution and lack of homogeneity of variances within the data.

### Video trials

To measure the impact of the different tomato species on the settling and movement behaviour of the whiteflies, video trials were conducted where a female whitefly was placed on the terminal leaflet of the tomato and its movement was recorded using a high-definition video camera (Sony HD Handycam, HDR-CX130). The whitefly was placed on the underside of the second apical leaf in a clip cage with a piece of transparent perforated plastic over the bottom, with the clip cage constructed so that the whitefly could leave the underside of the leaf, but was not able to reach the topside, only the sides and bottom of the clip cage. A recording of the whitefly was made for 65 min, which incorporated a 5-min period of recovery from the mild CO_2_ anaesthetisation used to capture and select the whitefly, and 60 min of data recording. This experiment was conducted for each of the tomato species under study, with 24 replicates per species. The data were analysed for four sets of behaviour over the 60 min: (1) time first present on the leaf (after 5 min recovery period), (2) time between the first and second stationary periods of >5 s on the leaf, (3) time spent moving and (4) time spent on the leaf. Comparisons were made between both tomato species using the Mann-Whitney *U* test due to deviations from the normal distribution and lack of homogeneity of variances within the data.

### Electrical penetration graph studies

To investigate the feeding behaviour of whitefly on *S. lycopersicum* Mill., ‘Elegance’ and *L. pimpinellifolium*, the electrical penetration graph (EPG) technique was used, as developed by Tjallingii (1978) and used previously to investigate whitefly feeding behaviour on tomato (Tosh and Brogan 2015; Lei et al. 1997, 1999, 2001). The different waveforms produced by the completion of a partial electrical circuit between the plant and the whitefly’s stylet when the whitefly probes the plant correspond to different feeding behaviours of the whitefly. An eight-channel DC EPG system supplied by EPG Systems (EPG-Systems, Dillenburg 12, 6703 CJ Wageningen, the Netherlands, http://www.epgsystems.eu/contact.htm; Tjallingii (1978)) was used. Tosh and Brogan (2015) developed a modified EPG apparatus (Fig. 2b, c) to monitor whitefly feeding under different conditions, and here, the same experimental set-up and method for the EPG data collection were used for both tomato species. Briefly, a single female whitefly attached with gold wire to the EPG apparatus was placed on the terminal leaflet of a tomato plant and allowed to feed for 20 h. Four replicates were run simultaneously (with one whitefly per plant for each of four plants). EPG waveforms for *L. pimpinellifolium* were collected, analysed and compared with waveforms previously collected by Tosh and Brogan (2015) for whiteflies feeding on the commercial *S. lycopersicum* cultivar ‘Elegance’ (unpublished data). In total, waveforms from 20 whiteflies feeding on *L. pimpinellifolium* and 23 whiteflies feeding on ‘Elegance’ were analysed. To identify the waveforms produced, the waveform guide supplied by Giga 4/8 EPG systems manual (http://www.epgsystems.eu/files/aphid%20waveforms.pdf) as well as two studies investigating whitefly-specific waveforms (Lei et al. (1997) and Lei et al. (1999)) were used. The waveforms of interest which may be observed on a whitefly EPG recording are C waveforms which indicate apoplastic stylet penetration and salivation, pd or potential drops indicating brief (4–12 s) intracellular probes and E waveforms indicating phloem penetration. The E waveforms may be divided into E1, indicating salivation into the phloem, and E2, indicating phloem sap ingestion. A probe is when an insect’s stylet is inserted into the plant. A “non-probe period” is when no waveform is observed due to an insect’s stylet being outside the plant (Rodríguez-López et al. 2011). Analysing the quantity, frequency and distribution of these waveforms during the EPG, both alone and in relation to each other, generates a large number of parameters which may be analysed to dissect insect feeding patterns. The raw data from the waveform analysis were exported to and analysed using the spreadsheet devised by Sarria et al. (2009).

## Results and discussion

Commercial tomato species exhibit a reduced ability to cope with attack by a wide range of pests and pathogens due to extensive selection through history (Sim et al. 2011). An effective strategy for increasing the resistance of commercial food crops is the introduction of genes that confer enhanced resistance to a target pest, as has been achieved for several insect/crop systems such as *B. tabaci* and cassava (Carabali et al. 2013). With this in mind, the current work aimed to assess the wild tomato species, *L. pimpinellifolium*, for resistance to *T. vaporariorum* and to attempt to identify the location of this resistance in the plant.

### Free choice assays

**Fig. 3:**
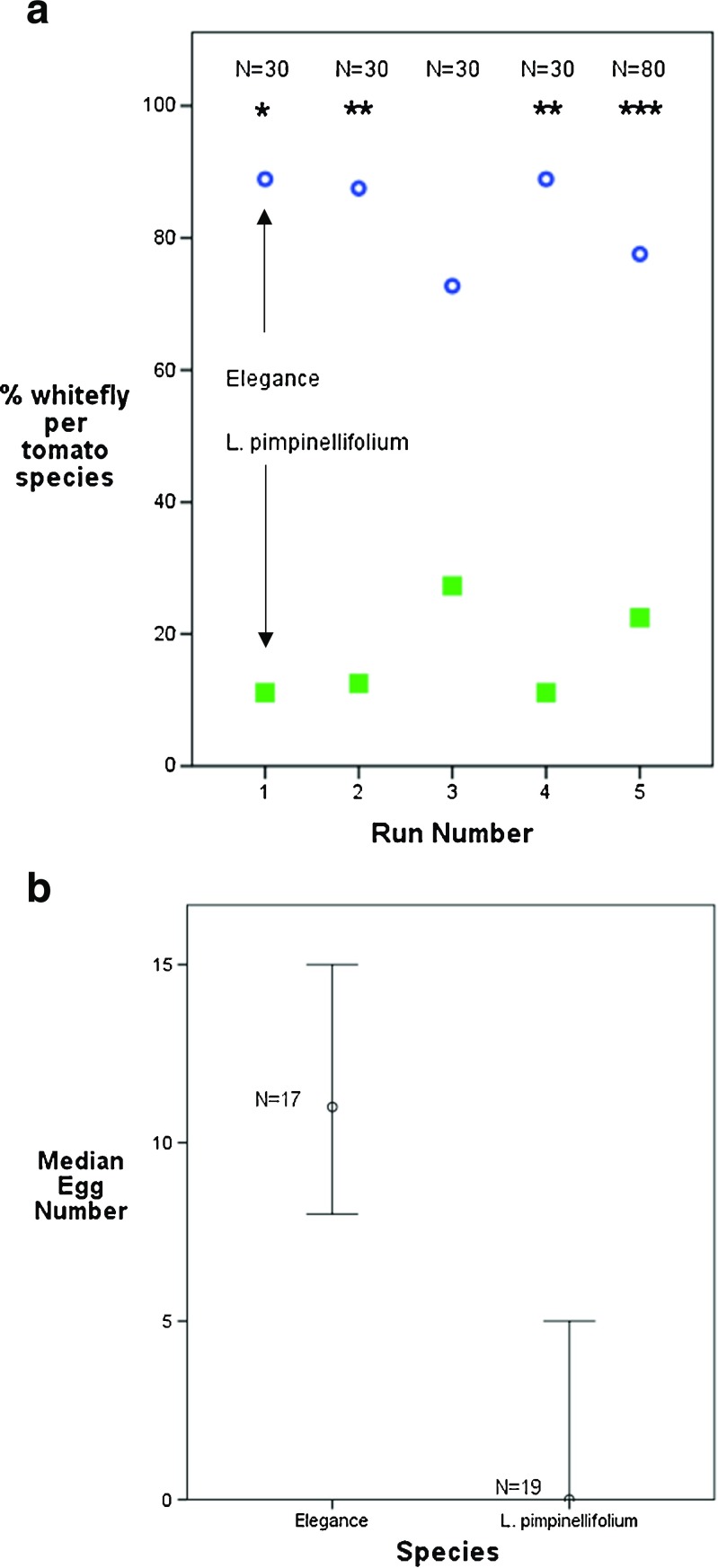
**a** The percentage of whiteflies settling on three plants of the commercial tomato species ‘Elegance’ and three plants of the wild *L. pimpinellifolium* after 24 h, repeated five times. Thirty whiteflies were used in rep nos. 1–4, 80 whiteflies were used in rep no. 5. *p = <0.05 significance; **p = <0.01 significance; ***p = <0.001 significance. *No asterisk* indicates a non-significant difference between the numbers of whitefly found on each species. Rep 1 *Χ* ^2^ = 4.00, *p* = <0.05; rep 2 *Χ* ^2^ = 7.56, *p* = <0.01; rep 3 *Χ* ^2^ = 3.68, *p* = >0.05; rep 4 *Χ* ^2^ = 9.39, *p* = <0.01 and 80 whiteflies in rep 5 gave *Χ* ^2^ = 13.80, *p* = <0.001, df = 1 for all reps. **b** The median number of eggs laid by a single female whitefly after 72 h on the second apical leaf of either ‘Elegance’ or *L. pimpinellifolium*. The difference is significant according to the Mann-Whitney *U* test with a *p* value <0.001. Ninety-five percent confidence intervals are shown. Test statistic, *U* (df = 34) = 28.5

When 30 whiteflies were given free choice between the commercial ‘Elegance’ and the wild *L. pimpinellifolium* over 24 h (Fig. 3a), a significantly higher number of whiteflies were found to settle on the commercial ‘Elegance’ compared to the wild *L. pimpinellifolium* according to Pearson’s chi-squared test. Similarly, when greater numbers of whitefly (80) were used, a highly significant preference (*p* < 0.001) for the commercial tomato species was observed (Fig. 3a). In all cases, more whiteflies were found on the commercial species than the wild tomato, with between 73 and 89 % of whitefly preferring to settle on the commercial vs. the wild tomato over the five trials. These data suggest a preference by the whitefly for the commercial species potentially because it represents a better food source due to a lack of resistance mechanisms that were present in the wild species. It deserves note that statistical analysis from run 1 had an inflated probability of a type 1 error, due to more than a fifth of the expected values equalling <5. However, as statistical output matched that from the other four runs (where this assumption was not violated), results of run 1 were retained and included herein. Firdaus et al. (2012) examined a range of wild tomato relatives for resistance to *B. tabaci*. They found that *L. pimpinellifolium* showed little evidence of being less preferable to *B. tabaci* based upon free choice assays, which is in contrast to the findings presented here. This may be due to differences in experimental design or be indicative of subtle differences in the ecology of the two whitefly species. Rodríguez-López et al. (2011) also conducted free choice assays in their comparison of the commercial tomato ‘Moneymaker’ and the ABL14-8 tomato breeding line, formed by the introgression of a *Solanum* *pimpinellifolium* L. accession into the ‘Moneymaker’ cultivar. They found that *B. tabaci* showed a strong preference for the commercial ‘Moneymaker’, similar to the identification of a preference for ‘Elegance’ found in the current study, but only in older plants (10-leaf vs. 4-leaf stage). The emergence of a stronger defensive response at an earlier stage in the present study may demonstrate a stronger presence of defensive traits in *L. pimpinellifolium* than in the ABL14-8 breeding line used by Rodríguez-López et al. (2011).

### No choice assays to record whitefly oviposition

The level of oviposition by the whitefly on ‘Elegance’ and *L. pimpinellifolium* after 72 h was analysed using the Mann-Whitney *U* test (Fig. 3b). A significantly greater number of eggs were laid on ‘Elegance’ compared to *L. pimpinellifolium* plants after 72 h, *p* < 0.001. These results demonstrate that the commercial tomato is a more preferred host for oviposition than the wild species. Bas et al. (1992) studied oviposition rates of *T. vaporariorum* on four genotypes of *Lycopersicon esculentum* varying in their resistance to the glasshouse whitefly and on two wild tomato species. The wild tomato *Lycopersicon hirsutum* var. *glabratum* was found to be most resistant and experienced the lowest oviposition rate. Whilst no resistance mechanism was suggested, the presence of greater resistance in the wild tomato species is concordant with our findings. Bas et al. (1992) also found that older individuals of *L. hirsutum* var. *glabratum* used in the study (those tested at 14 weeks rather than 8) displayed enhanced resistance, which has interesting implications for the present study in that the apparent resistance observed in *L. pimpinellifolium* in the present work at the 3–4 leaf stage (∼3 weeks old) may increase as the plants age. Erb et al. (1994) studied the potential of another wild tomato species, *Lycopersicon pennellii*, to act as a source of resistance traits against *T. vaporariorum*. Hybrids produced using this species supported the fewest eggs and were the least attractive hosts of the whitefly. Firdaus et al. (2012) also considered oviposition and found one accession of *L. pimpinellifolium* to be resistant on the basis of supporting low levels of egg-laying, as corroborated by the present work.

### Whitefly movement trials

**Fig. 4:**
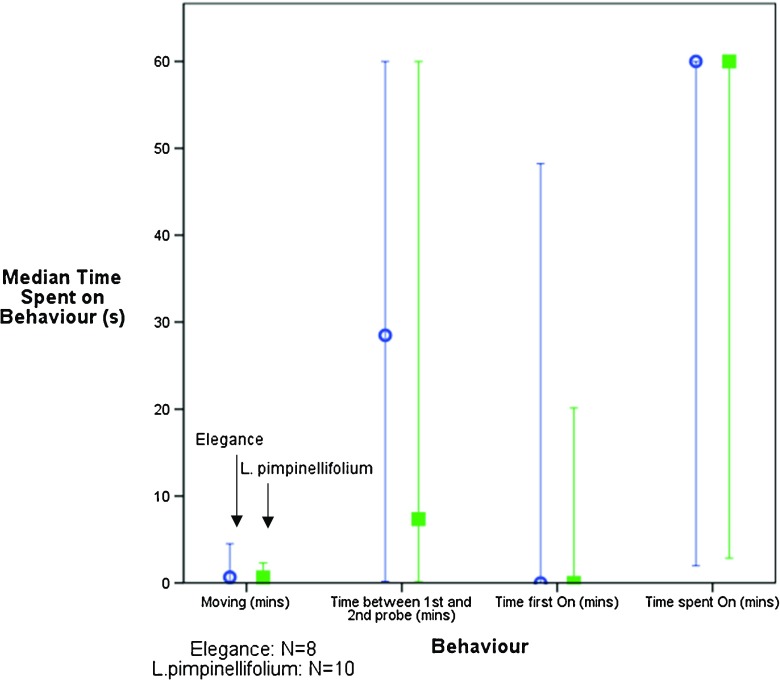
Different movement behaviours by a single whitefly on either the commercial ‘Elegance’ or the wild *L. pimpinellifolium* over 1 hour. The median and 95 % confidence intervals are indicated. Differences between the two species are non-significant. Test statistics for: Time first on: *U*, (df = 34) = 32 (*p* = 0.368); Time between first and second probe: *U*, (df = 34) = 35.5 (*p* = 0.684); Time spent moving: *U*, (df = 34) = 38.5 (*p* = 0.892) and Time spent on: *U*, (df = 34) = 39.5 (*p* = 0.958)

The median time spent by whiteflies engaging in selected behaviours on the two tomato species is shown in Figure 4. No significant differences between ‘Elegance’ and *L. pimpinellifolium* for any of the selected behaviours were observed. A large proportion of whiteflies on each tomato species (66 % for Elegance and 58 % for *L. pimpinellifolium*) did not land on the tomato leaves at all during the hour-long assay. This may represent a methodological limitation of the current study, and differences may have been detected had video capture periods been extended. Although these whiteflies were excluded from subsequent analysis, a chi-squared analysis revealed that there was no significant difference in the number of whiteflies which avoided the ‘Elegance’ or the wild tomato leaves compared to those which chose to land on the leaf (*Χ* ^2^ = 2, df = 1, *p* > 0.05, *N* = 24 for both). Many whitefly resistance mechanisms in tomato have been found to be surface based, including type IV trichomes which reduce whitefly feeding efficiency (Firdaus et al. 2012). Rodríguez-López et al. (2011) monitored the feeding behaviour *B. tabaci* on the commercial ‘Moneymaker’ strain of tomato and the ABL14-8 tomato breeding line. This breeding line was formed by the introgression of accession TO-937 of *Solanum pimpinellifolium* L. into the ‘Moneymaker’ cultivar and was backcrossed to exhibit a particularly high density of type IV trichomes and high acylsucrose production. This paper concluded that the presence of these surface-based resistance mechanisms deterred whitefly from landing and settling on the ABL14-8 breeding line. Lei et al. (2001) stated that the main resistance mechanism in the commercial ‘Moneydor’ species of tomato was the presence of very dense hairs on the leaf surfaces, physically preventing the whitefly from effectively probing. Erb et al. (1994) attributed the greater resistance of *L. pennellii* to *T. vaporariorum* to toxic exudates from glandular trichomes. Firdaus et al. (2012) also suggest that the resistance found in *L. pimpinellifolium* is based upon the presence of type IV trichomes. These studies contrast with the present work. The movement data presented here indicate that surface characteristics are not involved in the resistance of *L. pimpinellifolium* to whiteflies: the non-significant differences obtained for any whitefly behaviours on the leaf surface between the tomato species in this study would suggest that whiteflies easily navigate *L. pimpinellifolium* leaf surfaces. This discrepancy may be due to the methodological limitations mentioned above, with longer video capture periods possibly being needed to reveal the importance of trichomes on *L. pimpinellifolium*.

### EPG studies to monitor whitefly feeding behaviour

Whitefly feeding behaviour was analysed using the EPG technique, with parameter selection based on prior study by Lei et al. (1997), Lei et al. (1999), Lei et al. (2001) and Rodríguez-López et al. (2011). These parameters are detailed in Table 1 and are subdivided into those parameters which relate to pre-phloem probing behaviour and those which relate to phloem phase probing (similar to EPG work by Jiang et al. (2001)).

**Table 1:** Mean values and standard errors of electrical penetratin graph (EPG) parameters collected from *T. vaporariorum* probing the commercial tomato species ‘Elegance’ and the wild tomato species *Lycopersicon pimpinellifolium*. Replicate numbers (*N*) and *p* values according to the Mann-Whitney *U* test (or Pearson’s chi square for % whitefly entering E (phloem phase)) are indicated

	Elegance		*L. pimpinellifolium*		*p* value
Pre-phloem parameters					
1. Time to first probe from EPG start (min)	12.6 ± 4.5	*N* = 23	90.9 ± 48.4	*N* = 20	0.527
2. Duration of first probe (min)	14.1 ± 5.1	*N* = 23	2.0 ± 0.6	*N* = 20	0.080
3. Duration of second probe (min)	16.8 ± 4.8	*N* = 23	6.0 ± 3.7	*N* = 20	0.011
4. Total number of probes	25.3 ± 5.8	*N* = 23	31.75 ± 6.78	*N* = 20	0.575
5. Total number of C	31.3 ± 6.3	*N* = 23	32.7 ± 6.8	*N* = 20	0.961
6. Total duration of C (min)	410.2 ± 39.1	*N* = 23	193.8 ± 34.2	*N* = 20	0.001
7. Number of probes to the first E	10.4 ± 2.1	*N* = 19	17.8 ± 3.6	*N* = 13	0.092
8. Time from start of EPG to first E (min)	215.9 ± 35.9	*N* = 19	281.7 ± 42.4	*N* = 13	0.147
9. Time from first probe to 1st E (min)	206.0 ± 36.1	*N* = 19	243.8 ± 42.0	*N* = 13	0.270
10. Duration of np during first hour (min)	29.7 ± 3.5	*N* = 23	46.1 ± 3.8	*N* = 20	0.002
11. Duration of np during second hour (min)	29.4 ± 3.7	*N* = 23	40.3 ± 4.3	*N* = 20	0.062
12. Duration of np during third hour (min)	19.8 ± 3.8	*N* = 23	35.8 ± 5.3	*N* = 20	0.024
13. Duration of np during fourth hour (min)	28.7 ± 5.0	*N* = 23	35.5 ± 5.6	*N* = 20	0.581
14. Duration of np during fifth hour (min)	30.1 ± 5.1	*N* = 23	31.6 ± 6.2	*N* = 20	0.911
15. Duration of np during sixth hour (min)	28.2 ± 5.4	*N* = 23	29.3 ± 6.0	*N* = 20	0.667
Phloem parameters					
16. Number of E	6.4 ± 1.5	*N* = 23	8.5 ± 2.8	*N* = 20	0.796
17. Total duration of E (min)	95 ± 31.4	*N* = 19	323.7 ± 59.6	*N* = 13	0.002
18. Duration of first E (min)	22.0 ± 20.3	*N* = 19	26.0 ± 14.0	*N* = 13	0.024
19. Number of probes after first E	18.4 ± 5.4	*N* = 19	20.8 ± 6.5	*N* = 13	0.999
20. Total duration of E1 (min)	34.0 ± 9.0	*N* = 19	257.1 ± 57.9	*N* = 13	0.001
21. Number of E1	5.3 ± 1.2	*N* = 23	5.4 ± 1.7	*N* = 20	0.538
22. Total duration of E2 (min)	144.9 ± 56.2	*N* = 8	96.3 ± 42.5	*N* = 9	0.370
23. Number of E2	1.1 ± 0.5	*N* = 23	3.1 ± 1.2	*N* = 23	0.269
24. % whitefly entering E phase	82.6	*N* = 23	65.0	*N* = 20	0.186

*C* pathway phase probing, *E* phloem phase probing, *np* not probing

The differences between Elegance and *L. pimpinellifolium* for most of the parameters measured were found to be non-significant by the Mann-Whitney *U* test, although ‘Duration of First Probe’ (*p =* 0.080), ‘Number of probes to the First E’ (*p* = 0.092) and ‘Duration of np during second hour’ (*p* = 0.062) approached statistical significance (Table 1). However, several parameters did show significant differences. Of the pre-phloem parameters, the ‘Duration of the Second Probe’ (*p* = 0.01) and ‘Duration of C’, or pathway, probing (*p* = 0.001) were found to be significantly shorter in *L. pimpinellifolium* than in ‘Elegance’, and the ‘Duration of non-probing behaviour’ by the whitefly in the first and third hours of the probe (*p* = 0.002 and 0.024, respectively) was found to be significantly longer in the wild species compared to the commercial species. Of the phloem-based parameters, the ‘Total Duration of E’, or phloem, probing (*p* = 0.002), ‘Duration of the first E probe’ (*p* = 0.024) and the ‘Total Duration of E1’, the waveform indicating sieve element salivation (*p* = 0.001), were significantly longer in *L. pimpinellifolium* compared to ‘Elegance’. These results indicate that whiteflies encounter difficulties when feeding on *L. pimpinellifolium* compared to ‘Elegance’. EPG studies have also been conducted by other authors on tomato species differing in *T. vaporariorum* susceptibility. Two such studies are Lei et al. (1999, 2001). In these experiments, the commercial ‘Moneymaker’ and two resistant lines (produced using *L. hirsutum glabratum* as a resistance source and named the 82216 and the 82207 resistant lines) were compared. In the study by Lei et al. (1999), it was proposed that the primary mechanism of resistance for line 82207 was located in the phloem sap. This was supported by the difference between the commercial and resistant lines in a number of EPG parameters, including a significantly higher total number of phloem phases, a shorter initial phloem phase, a longer phloem subphase 1 (E1) and a shorter subphase 2 (E2) that were found in the resistant 82207 line compared to the ‘Moneymaker’ line. The EPG data presented here support a longer E1 phloem subphase as being linked to resistance, though differences in other parameters identified as important by Lei et al. (1999) were not detected. Differences in other parameters were nevertheless detected in the current work (‘Duration of second probe’, ‘Duration of C’ and ‘Duration of np in the first and third hours) that may indicate the presence of a resistance mechanism in a different location to that found in line 82207. Rodríguez-López et al. (2011) showed that *B. tabaci* is less able to reach the phloem, spent more time in non-probing activities and displayed a reduced amount of probing on the ABL14-8 tomato breeding line than the commercial ‘Moneymaker’. The present study supports the finding of more non-probing behaviour but only over the first 3 h, after which the effect disappears. The results of the present study also indicate no difference in the ability of *T. vaporariorum* to access the phloem of either *L. pimpinellifolium* or ‘Elegance’. These differences may occur as a result of differences in the whitefly species used, revealing subtle differences in the ecology of these two species, or as a result of the differences in the wild tomato species used.

### Proposed location of resistance to *T. vaporariorum* in *L. pimpinellifolium*

Based upon these data, it is proposed that two separate resistance factors are present in *L. pimpinellifolium*: a resistance factor encountered early during *T. vaporariorum* feeding and a phloem-based resistance factor. The first resistance factor is proposed to be epidermal/mesophyll-based, encountered by the whitefly during labial dabbing as it assesses the tomato as a prospective host and during pathway probing as the whitefly attempts to locate the phloem. It has long been known that phloem-feeding insects conduct shorter, gustatory sampling probes at the start of a feeding bout in order to assess the quality of the host plant (Tosh et al. 2002). When using the EPG technique to study aphid plant probing, gustatory sampling is indicated by an abundance of potential drops on the trace (Tjallingii 1985) which indicate the puncturing of host plant cells. However, whitefly have been shown to be less invasive in their feeding method, moving their stylet between cells rather than puncturing them to sample the internal contents (Lei et al. 1997). This indicates that any sub-epidermal resistance mechanism is unlikely to occur inside cells punctured en route to the plant phloem and instead is located extracellularly in the mesophyll. This pre-phloem-based factor is further supported by the significantly shorter ‘Duration of the second probe’ and ‘Duration of C waveforms’ (pathway-phase probing) and the significantly higher level of non-probing behaviour in the early part of the EPG trace. It is suggested that the whitefly encounters this factor during its initial probe and that this factor causes the whitefly to attempt to avoid this aversive element by reducing the length of its next probe, spending less time in the mesophyll of the plant and spending more time in non-probing behaviours, e.g. resting or moving to avoid the resistance factor. This is also supported by the free choice data, where a significant proportion of the whiteflies had moved to the more palatable ‘Elegance’ tomato variety over the course of the experiment. Whilst we have not directly observed the timing of this movement between the tomato species, the increased restlessness of the whitefly in the first 3 h of the EPG experiments (indicated by the significantly greater level of non-probing) suggests that the whitefly respond quickly upon exposure to this mesophyll-based mechanism.

During the EPG experiment, the whitefly were tethered and therefore forced to interact with and feed upon the wild tomato species to avoid starvation. This may account for the lack of a significant difference in the non-probing behaviours 3–6 h after the start of the experiment. As can be seen by the duration of E2 probing, after 15 h, there was no significant difference in the amount of time spent ingesting the contents of the phloem presumably because the whitefly must feed to avoid starvation due to this forced interaction. This pattern is repeated in the findings of studies into the mode of action of the *Mi-1.2* gene, which confers resistance to nematodes, aphids and whitefly in tomato. Jiang et al. (2001) suggested that the *Mi-1.2* gene was expressed in the mesophyll or epidermis of the plant and that when the whitefly had free choice they avoided tomatoes possessing *Mi-1.2*. When they were forced to interact with the plant, however, they were able to access the phloem in a similar manner to the control. Whilst we cannot claim to have discovered *Mi-1.2* in *L. pimpinellifolium*, as this gene originated from a different wild tomato species (*Lycopersicon peruvianum*), the similar mode of action of the resistance mechanism described may suggest evolution of a similar gene to deter whitefly in *L. pimpinellifolium*. This lends credence to our suggestion that a similar factor may be present in this wild tomato species.

The second resistance factor proposed is a phloem-based factor. This is evidenced by the significantly higher level of E1 phase probing, or salivation, of the whitefly when accessing the phloem of *L. pimpinellifolium*. Salivation occurs in order to prepare the phloem for whitefly feeding and as such takes up a relatively small proportion of the phloem phase when the whitefly is feeding on a susceptible host. The significantly greater amount of salivation, and length of the first E probe (likely to be an E1 probe), observed when *T. vaporariorum* fed on *L. pimpinellifolium* is therefore indicative of a less favourable interaction between the insect and the host. It has long been hypothesised that aphids produce watery saliva during feeding to combat occlusion of the phloem sieve elements (e.g. Tjallingii and Esch (1993)) and evidence for this is provided by elegant work using legume forisomes (Will et al. 2007). That whiteflies utilise the same method has also been suggested in work by Liu et al. (2013). The authors reported that *B. tabaci* infected with *Tomato yellow leaf curl virus* showed extensive salivation into sieve elements, which was interpreted as the virus affecting the ability of the whitefly to prevent sieve element occlusion to enhance the virus’ transmission in watery saliva. In both whitefly and aphids, increased or extended salivation has been correlated with feeding on resistant plants (Will et al. (2007); Jiang and Walker (2007)). Sieve element occlusion is a mechanism employed by most plants to prevent loss of sap from ruptures in the phloem. It is also employed as a resistance mechanism against phloem-feeding insects. Aphid watery saliva contains proteins which bind to calcium and prevent the signalling cascade leading to occlusion of sieve elements by the plant (Will et al. 2007). Recent analysis of whitefly salivary glands has revealed that these glands contain genes encoding several calcium-binding proteins which are hypothesised by the authors to fulfil the same function in whitefly saliva as in saliva of aphids (Su et al. 2012). It is therefore possible that whitefly saliva is able to prevent sieve element occlusion and allow continued access to the phloem. The greater amount of salivation observed in this work is therefore suggested to be a response by *T. vaporariorum* to a much stronger defensive effort by *L. pimpinellifolium* to plug the holes in the phloem than was exhibited by the commercial tomato. The significantly greater amount of salivation by *T. vaporariorum* when feeding on *L. pimpinellifolium* is proposed to account for both the greater “Total duration of E” (as E phase probing comprises total time of E1 and E2 waveforms, and E2 showed no significant difference between the tomato species) and the greater length of the first E probe (as greater salivation was required for a successful phloem-phase probe). The increased level of salivation required to successfully access the phloem of *L. pimpinellifolium* may indicate that the wild species possesses genes which allow it to mount this more effective response, which are attractive targets for incorporation into the ‘Elegance’ genome. Whilst there was no difference in the amount of E2 waveforms, showing that the glasshouse whitefly is able to ingest as much sap from *L. pimpinellifolium* as it does from ‘Elegance’, the increased effort and energetic expenditure required to do so may be sufficient to deter feeding in a situation where the whitefly has the choice to move onto a less-resistant host. This evidence of a sub-epidermal source of resistance and a phloem-based resistance mechanism provide interesting targets for breeding programmes to attempt to incorporate into commercial tomato species.

Future research should focus on the genes, or sets of genes, which confer the two resistance mechanisms suggested here. The introduction of these whitefly resistance genes could potentially aid the continued and more effective production of tomato plants in the future. Future work could also involve determining whether whiteflies definitely obtain gustatory information about a plant during an initial probe. This would confirm the suspected sub-epidermal location of the resistance mechanism of *L. pimpinellifolium*.

## Conclusion

The wild tomato species *L. pimpinellifolium* represents a source of genetic resistance to *T. vaporariorum*, based on the oviposition and settling behaviour of whitefly on the wild species when compared to the commercial tomato variety *S. lycopersicum* ‘Elegance’. The resistance in *L. pimpinellifolium* appears to be based upon a dual mechanism: a post-penetration but pre-phloem resistance mechanism similar to the *Mi1.2* gene previously discovered in other species of tomato and a phloem-based mechanism which may be linked to sieve element occlusion. It is hoped that this work describing resistance in *L. pimpinellifolium* will inform future breeding programmes for the introduction of whitefly resistant genes into commercial varieties of this highly important crop plant.
